# Selection strategy and the design of hybrid oligonucleotide primers for RACE-PCR: cloning a family of toxin-like sequences from *Agelena orientalis*

**DOI:** 10.1186/1471-2199-8-32

**Published:** 2007-05-11

**Authors:** Zhensheng Pan, Richard Barry, Alexey Lipkin, Mikhail Soloviev

**Affiliations:** 1 School of Biological Science, Royal Holloway, University of London, Egham, Surrey, TW20 0EX, UK; 2 Shemyakin & Ovchinnikov Institute of Bioorganic Chemistry, Ul. Miklukho-Maklaya, 16/10, 117997 GSP, Moscow V-437, Russia

## Abstract

**Background:**

the use of specific but partially degenerate primers for nucleic acid hybridisations and PCRs amplification of known or unknown gene families was first reported well over a decade ago and the technique has been used widely since then.

**Results:**

here we report a novel and successful selection strategy for the design of hybrid partially degenerate primers for use with RT-PCR and RACE-PCR for the identification of unknown gene families. The technique (named PaBaLiS) has proven very effective as it allowed us to identify and clone a large group of mRNAs encoding neurotoxin-like polypeptide pools from the venom of *Agelena orientalis *species of spider. Our approach differs radically from the generally accepted CODEHOP principle first reported in 1998. Most importantly, our method has proven very efficient by performing better than an independently generated high throughput EST cloning programme. Our method yielded nearly 130 non-identical sequences from *Agelena orientalis*, whilst the EST cloning technique yielded only 48 non-identical sequences from 2100 clones obtained from the same *Agelena *material. In addition to the primer design approach reported here, which is almost universally applicable to any PCR cloning application, our results also indicate that venom of *Agelena orientalis *spider contains a much larger family of related toxin-like sequences than previously thought.

**Conclusion:**

with upwards of 100,000 species of spider thought to exist, and a propensity for producing diverse peptide pools, many more peptides of pharmacological importance await discovery. We envisage that some of these peptides and their recombinant derivatives will provide a new range of tools for neuroscience research and could also facilitate the development of a new generation of analgesic drugs and insecticides.

## Background

Toxins and toxin-like molecules are present and used widely throughout the animal kingdom. Among the arthropods, which constitute over 90% of the animal kingdom and include bees, wasps, ants, spiders, scorpions and many other various taxa, many are well known for their predacity and toxic venoms. These have evolved to yield complex and highly specialised toxins which are now successfully used by these predaceous animals to either protect themselves or attack their prey. Despite being considered the most successful animals on Earth (over one million species known and up to 6–9 million species predicted to exist in total) and the massive research effort so far, only a tiny proportion of arthropods has been studied in detail. Spiders evolved from an arachnid ancestor around 400 million years ago and currently comprise over 100,000 different species. Spiders are the most successful predatory animals, in evolutionary terms, and they maintain by far the largest pool of toxic peptides. There are over 39,000 catalogued species [1], with an even larger number still awaiting characterisation [2]. It has been calculated, based upon a conservative estimate of some 80,000 species and approximately 50 peptides per species [3], that there are in the region of 4 million distinct spider-venom polypeptides in existence [4] although of these only a few venoms have been characterised. Only a few hundred of known toxins or toxin-like genes have so far been reported (worldwide) from arthropods or other venomous creatures such as snails or snakes. The precise composition of spider venoms varies significantly between different species. Spider toxins are thought to have derived from a small number of gene super-families with many peptide toxins sharing structural features, conserved amino acids and consensus sequences. This allows them to interact with specific targets such as related classes of cellular receptors. The wide array of peptides may be associated with spiders being general predators, i.e. they do not focus on one specific prey species. It has been suggested that the generation of peptide toxin diversity in spiders has probably been achieved via a similar process to that of cone snails (a group of predatory marine snails which produce an array of neurotoxic peptides collectively named conotoxins), i.e. via extensive gene duplication followed by key hyper-mutations of the pro-peptide and mature-toxin segments. The resultant pool of genes has subsequently been subjected to the pressures of adaptive evolution and this has culminated in the vast arrays of species-specific combinatorial peptide libraries which now exist [4]. It is by the very nature of these processes that an opportunity to discover new peptides of pharmacological importance has become possible using techniques such as PCR-based cloning incorporating degenerate primers, and EST-based cloning.

One of the few successful "molecular" approaches to molecular cloning of polypeptide toxins was reported recently by Kozlov *et al*. [5,6]. There an EST (expressed sequence tag) high throughput cloning strategy was employed which yielded nearly 50 novel toxin and toxin-like sequences from *Agelena orientalis *following the sequencing of 2166 individual EST clones [6]. There are two main advantages to using the EST based approach: the discovery of novel genes and their mRNAs/cDNAs does not require any prior knowledge of the nucleic acid or amino acid sequence information (open system) and if applied to non-normalised cDNA libraries (or pools) it often yields quantitative information on the individual transcripts abundance. This allows, at least in principle, the discovery and expression profiling of all genes expressed in the cell/tissue/etc. without any pre-selection. This useful property is widely relied upon in pharmacogenomics, drug discovery, biomedical and plant sciences (for reviews see [7-13]). For the same reasons, and unless an EST cloning approach is used to explore fully normalised cDNA libraries, the outcome will be biased towards the highly abundant transcripts whilst lower abundance molecular species will likely escape detection. Not surprisingly, EST sequencing of *Agelena orientalis *revealed that over 70% of all of the EST clones sequences (1497 out of 2166) encoded the same transcript Agelenin (see [6]), whilst most of the other newly identified genes (47 altogether) were represented by just a few or in most cases by a single EST [6]. EST based cloning is a generic and universal approach to the discovery and expression profiling of genes, but despite its wide applicability it has a few drawbacks, such as the above discussed bias towards highly abundant transcripts and the requirements for lab automation, which are not universally available. This chapter aims to illustrate an alternative approach to the discovery of novel families of toxin or toxin-like polypeptides.

**Figure 1:**
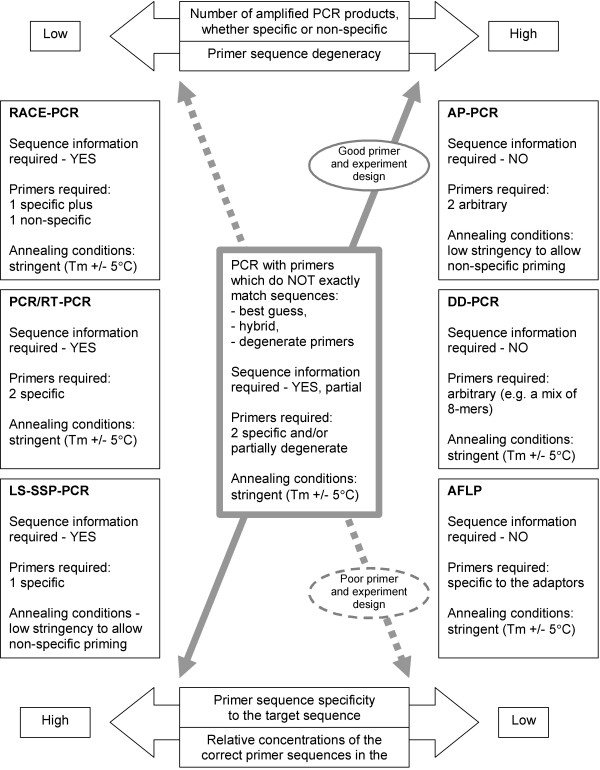
**Overview of PCR-based strategies suitable for the amplification of cDNAs**. For references see PCR/RT-PCR [14, 15], RACE-PCR [19], LS-SSP-PCR [23], AP-PCR/DD-PCR [24, 25] and AFLP [26]. The use of nested primers not shown as the principle which can be applied to any of the above or their combinations. A PCR amplification relying on the use of fully degenerate, partially degenerate (hybrid) or "best guess" primers occupies a place in-between the PCR approaches illustrated. The outcome of such PCR may vary, as schematically indicated in grey lines. The best case scenario indicated with solid grey line, the worst case scenario – with dashed grey line.

PCR amplification [14,15] has revolutionised life sciences [16,17] and with all its numerous variations has become one of the most widely used techniques in molecular biology. PCR has been described elsewhere; see e.g. [18] and references therein. The main PCR variations, related to cDNA amplification and cloning include PCR/RT-PCR, RACE-PCR [19-21] and the related RLM-RACE-PCR [22]; LS-SSP-PCR [23], AP-PCR/DD-PCR [24,25] and AFLP [26], see Figure 1(these do not include PCR with nested primers [27], of which the principle can be applied to any of the above or their combinations). The former three PCR processes have one important feature in common – they all require the knowledge of the exact sequence on which the oligonucleotide primer(s) are based. The use of the PCR/RT-PCR, RACE-PCR and to a lesser degree LS-SSP-PCR, results in the amplification of one or a few target-specific sequences situated between the two primer annealing sites (PCR, RT-PCR), between the primer annealing site and 5'- or 3'- ends (RACE-PCR), or between the specific primer annealing site and a few non specific annealing sites on the same DNA as in LS-SSP-PCR. Unlike these, AP-PCR/DD-PCR and AFLP do not require any knowledge of the target sequence (are open systems). However, these methods typically result in the amplification of a much larger number of DNA fragments and are generally most useful for various DNA fingerprinting applications. Block arrows (Figure 1, top and bottom) summarise the above PCR strategies by indicating the overall trends in primer sequence specificity vs. sequence degeneracy and vs. a typical number of amplified products.

The use of specific but partially degenerate or hybrid primers for nucleic acid hybridisations and PCRs amplification of known or unknown gene families was first reported well over a decade ago (see e.g. [28] for the use of degenerate primers in hybridisations and [29,30] for PCR applications) and further mastered by Rose *et al*. (see [31,32] and references therein). The technique has been used widely since then. Introducing degeneracy into the primer sequence aims to compensate for codon degeneracy (if only the amino acid sequence is known) or amino acid degeneracy (if the aim is to amplify a family of DNAs encoding proteins having multiple amino acids at a position in the alignment). The most common problems of degenerate primer design include:

**i. **too high a degeneracy leading to elevated non-specific amplification (due to an abundance of irrelevant sequences) and absence of specific amplification (due to low relative concentration of the correct primer sequence and early depletion of that pool)

**ii. **too high a degeneracy leading to the formation of secondary structures and self-annealing, detrimental to a PCR amplification

**iii. **high variability in the Tm within the pool of degenerate primers, making it impossible to choose optimal annealing temperatures

**iv. **a further potential problem of the approach is the inability to define suitable regions of high sequence conservation and thus an inability to design any primers at all, e.g when the sequences analysed are too dissimilar and/or when a large number of sequences are being aligned (often resulting in no homologous regions altogether).

Both primer design [31-33] and the choice of conserved sequence fragments are key issues for the success of any such amplification. Although easy in principle, practical applications of degenerate primers are often limited to the amplification of gene/protein families having a high degree of sequence similarity and containing low degeneracy codons in the positions corresponding to the PCR primers. Successful application of degenerate or hybrid primers also depend on the particular application, target abundance and target sequence(s) variation. A PCR/RT-PCR employing partially degenerate primers occupies a place somewhat in-between the other PCR approaches mentioned above, see (Figure 1) and its applications range from the use of low degeneracy "best guess" primers (allowing typically for a more specific annealing and amplification) to hybrid or even fully degenerate primers which have a tendency to amplify more fragments, which are often more non-specific fragments, and to a lower degree. The range of PCRs with degenerate primers is schematically indicated on Figure 1 with grey arrows.

## Results and discussion

### Multiple alignment of a large number of existing toxin and toxin-like sequences yields no universal consensus sequence

**Figure 2:**
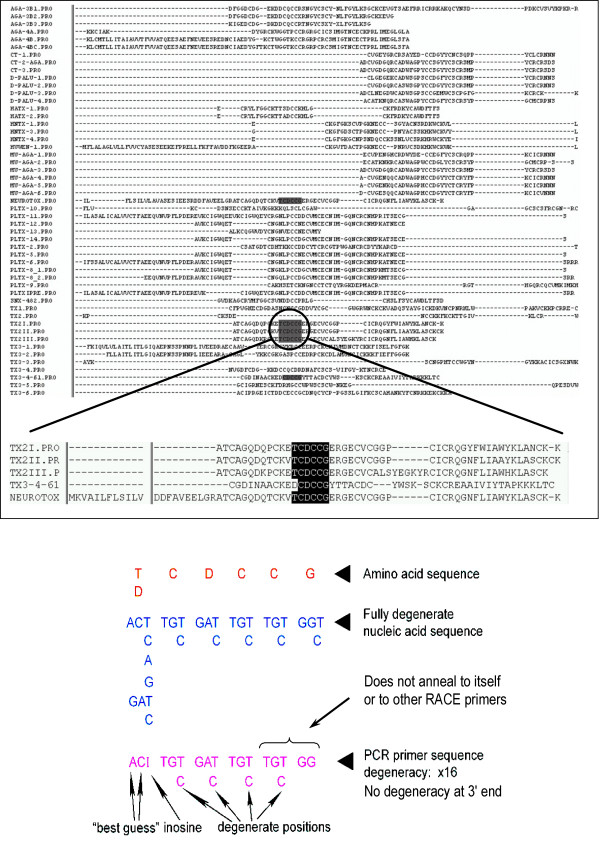
**Multialignment of the protein toxin sequences obtained form Genbank and SwissProt**. Over 150 toxin and toxin-like sequences were available in the beginning of investigation and were aligned to identify potential conservative sequence motifs. 51 sequences shown aligned (top panel). The five sequences (TX2I, TX2II, TX2III, TX3-4-61 and NEUROTOX from *Phoneutria nigriventer *(Brazilian armed spider)) selected to exemplify a conservative motif "T/DCDCCG". Bottom panel exemplifies the strategy of primer design.

The starting point of this investigation was the realisation that the number of known toxin and toxin-like peptides (from arthropods and alike) was in excess of a hundred (based on a PubMed search at the beginning of this work). It was also not unreasonable to assume that the target species *Agelena orientalis *could have contained more than just a few novel related toxin-like sequences. Our approach to the problem of cloning a potentially large peptide family of toxin-like peptides consisted of a number of logical steps, key enabling assumptions and technical solutions which eventually led to the identification of over 100 distinct cDNAs, and which can be applied to any similar problem of identifying large families of expressed peptides or proteins. The first step was to align the existing toxin and toxin-like sequences (over 150 sequences were aligned in total). This was in order to find regions with high sequence homology or conservation. Unfortunately this resulted in that *no single universal consensus sequence was identified*. This was not unexpected and was also in agreement with the reports on cone snails by Duda & Palumbi (in which the repertoire of mature toxin sequences contained non-conservative substitutions in nearly all positions except for the cysteine residues [34]) and, more recently, on arthropods by Kozlov *et al*. (where the alignment of ca. 300 arthropod toxin sequences yielded no conservative regions [6] and hence no easy chance to design a universal consensus primer). However a number of short consensus regions were indeed identified between *small subsets *(ca 2–5 individual sequences on average, see e.g. Figure 2). Identical or highly similar sequence fragments found in at least 3 different protein toxins were considered for further analysis.

### The design of hybrid partially degenerate primers

Short subsets of toxin and toxin-like sequences, regardless of their origin (i.e. species), sequence/structure (i.e. short/long, cysteine pattern, presence of any secondary structure elements) or function (i.e. functional toxin or simply toxin-like sequence), were considered but only if they resulted in any alignment suitable for primer design. Although sequence similarities between the small number of highly related sequences are not likely to identify real consensus sequences the use of these was often the only possibility. However, trying hundreds of such fragments would be impractical. Therefore only those were chosen which satisfy the criteria listed below.

**Figure 3:**
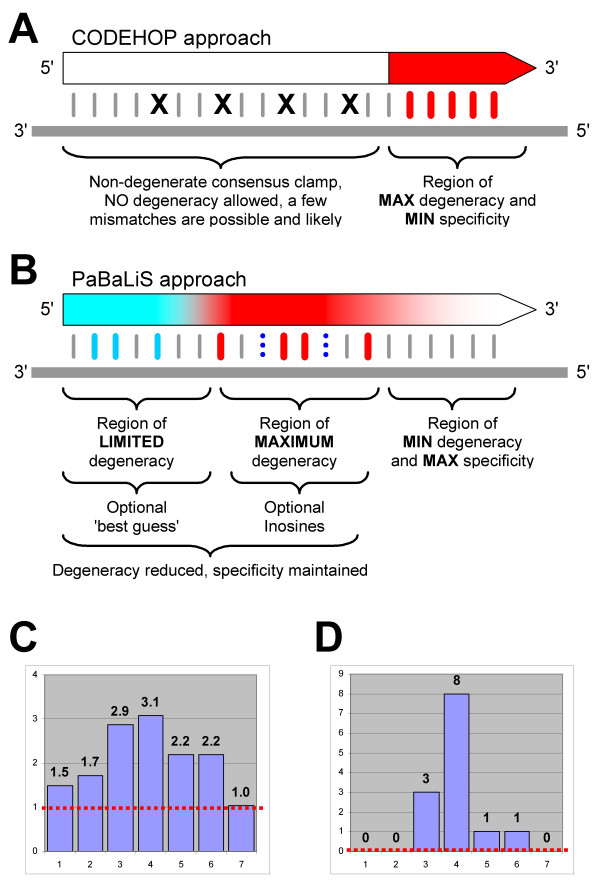
**The comparison of CODEHOP and PaBaLiS primers**. (A) Schematic representation of the CODEHOP primer, based on the original design by Rose *et al*. [31]. The maximum degeneracy is aimed at the 3' region, which reduces the amplification efficiency (due to competition by "wrong" 3' end sequences for annealing, these will disallow amplification) and very high probability of dimer formation by such oligos (e.g. fully degenerate 3-4-5 or 6-mers will all able to dimerise). (B) Schematic representation of the PaBaLiS primer design. Note that maximum degeneracy is achieved in the middle of the primer, minimising the number of possible mismatches in the middle of the primer and increasing the stability of the hybrid (primer/cDNA). Low degeneracy (and high sequence conservation) at the 3' end ensured the highest specificity of annealing, amplification and allows to easily avoid primer dimers. (C) The actual distribution of degenerate bases for PaBaLiS primers designed in this study. The choice of oligonucleotides for this analysis includes the 22 oligonucleotides of identical length (20-mer long oligonucleotides, based on 7 amino acid long stretches of partially of fully homologous protein sequences) of the 44 oligonucleotides designed in total. The horizontal axis indicated the nucleotide degeneracy in triplets approximately corresponding to each of the 7 amino acid positions used to generate these 20-mer oligonucleotides. The mean degeneracy calculated as total degeneracy at this position (due to both the amino acid and the genetic code degeneracy) divided by the number of oligonucleotides). The numbers of the bars indicate actual values. Note that decreased overall degeneracy at 5' end is due to the use of "best guess" based and triplets, but not because amino acid degeneracy was lower at this position. (D) The actual distribution of inosine substitutions in the same PaBaLiS primers as in (C). The use of inosines allowed to decrease overall degeneracy without introducing mismatches in the middle of the primers,

The original communication by Rose *et al*. [31] reported one of the first successful uses of hybrid partially degenerate primers for PCR detection of diverse reverse transcriptase-like genes from a human genome, and of detection of C5 DNA methyltransferase homologs from plant DNAs. Contrary to the report of Rose *et al*., in which the regions of sequence homology and lowest degeneracy were at the 3' end and the highest degeneracy was at the 5' end, we have chosen to *minimise or avoid completely any degeneracy at the 5' end *of the oligonucleotide primers. Figure 3 depicts the fundamentally different design principles between the CODEHOP approach [31] (Figure 3A) and our approach (Figure 3B), which was named PaBaLiS (after the authors' names) for brevity and in order to maintain a commonly followed tradition of meaningful naming. In the design by Rose *et al*. [31], the annealing of the sequence-specific non-degenerate consensus clamp at the 5' end to the template DNA strand does not guarantee that the correct 3' sequence is present in this primer. Therefore such 3' ends would compete with the correct 3' sequences and the efficiency of amplification will be reduced proportionally to the primer degeneracy. If highly degenerate primers are used (i.e. correct 3' end is present at low concentration compared to the arbitrary/wrong 3' end components), amplification will seize. Therefore 3' ends of PCR primers should ideally be non-degenerate to increase the specificity of amplification and efficiency of amplification, minimise non-specific priming, avoid forming primer dimers and provide a means to utilise even mismatched primers for amplification. As the PCR amplification progresses and the 100% matching primers are used-up, partially mismatching ones would become capable of priming PCR amplification at the later amplification steps, even having a few mismatches at the 5' end, but *not *at 3' end (as in CODEHOP approach). We have therefore limited our search to such homologous regions (of protein sequences) which could be used in the design of 3' low degeneracy primers (i.e. having low amino acid and codon degeneracy at their 3' end).

The next criterion was to allow the highest degeneracy in the middle of PCR primers (this was to guarantee 100 % sequence matching and hence the maximum hybrid stability). Furthermore, the 5' end of the oligonucleotide primers was often designed to have a "best guess" sequence (based on both amino acid degeneracy at the position and the codon usage frequencies). This often significantly reduced the overall sequence degeneracy, at a small price of possibly introducing mismatches at the very 5' end (these would not destabilise the hybrid compared to any mismatches in the middle of the primer). Figure 3C shows the very different distribution of degenerate bases for the 22 oligonucleotides of identical length (20-mers) out of the 44 oligonucleotides designed in this study. The maximum degeneracy is reached in the middle of the primer unlike the CODEHOP primers (Figure 3A).

The overall primer length was kept to an average of 20 bases (17–23 bases range) and the overall degeneracy – to below approx ~64. The latter is because higher degeneracy means a lower concentration of any single primer and their quick depletion from the pool of available primers as PCR amplification proceeds. Although the unique primer design allows us to utilise some partially matching primers to boost the effective concentration of the working primer, a degeneracy over ~100 was found to be detrimental in our previous experience. Therefore, in the regions where designed primers did not satisfy the degeneracy criteria, Inosine was substituted at the most highly degenerate positions between 5' end and approximately the middle of the oligonucleotide sequence. Figure 3D shows the actual distribution of Inosine residues for the 22 oligonucleotides of identical length (20-mers) out of the 44 oligonucleotides designed in this study. Figure 2 gives an easy to follow example of the primer design. Table 1 lists all the sequence specific primers designed, to allow the reader to appreciate the range of primer design outcomes.

We can summarise the key advantages of PaBaliS over CODEHOP as follows:

**i. **highest specificity of annealing and amplification due to low (or absence) of degeneracy at 3' end,

**ii. **less stringent requirements for the length and composition of the protein consensus sequence, which in PaBaLiS is at the 3' end and is generally shorter than the equivalent "non-degenerate consensus clamp" 5' region in CODEHOP.

**iii. **PaBaLiS design allows avoidance of dimer formation (through non-specific annealing) since 3' ends are unique non-degenerate sequences and can be easily designed not to dimerise: in contrast to CODEHOP, where all primers will find their complementary 3' pair and will tend to dimerise (leading to quick depletion of the primer pool, non specific priming and poor amplification).

**iv. **PaBaLiS primers have lower degeneracy overall, and even primers with a few mismatching positions will be able to anneal specifically and continue amplification at later stages of the amplification, when the 100% matching primers are exhausted

**v. **PaBaLiS primer design yields a larger fraction of primers with *no *mismatches at the 3' end (meaning that *all *primers have specific 3' ends, which is most important for the specificity and efficiency of amplification).

**vi. **PaBaLiS primers have *fewer *mismatches in the middle of the primer (since degeneracy is allowed) and therefore higher overall hybrid forming stability (unlike CODEHOP where mismatches are possible in the middle of the primer, thus destabilising the hybrids).

The limitations of the CODEHOP approach were highlighted in a recent report by Gray and Coates [35] where the authors had to use two completely nested sets of CODEHOP RT-PCR primers to amplify a highly-conserved 168 amino acid long region (only to design RACE primers for the next amplification round), whilst PaBaLiS primers apparently work directly for RACE-PCR and can be designed to amplify sequences with just a few amino acids homology, and which do not need to be between consecutive amino acids (small gaps allowed).

### Annealing temperature matching

The constraints described above reduced the number of suitable regions significantly in our case and would undoubtedly do so in any other similar cloning approach. One other important criterion of the primer design is in a reduction of the range of annealing temperatures for highly degenerate primers and matching these for the pairs of PCR primers. We soon realised that no suitable 5' and 3' PCR primer *pairs *(especially with the matching annealing temperatures) could be obtained. Therefore the decision was to opt for RACE-PCR. This is likely to be the only available option in any similar cloning project, when not enough sequence information is available for the design of more than one sequence-specific primer (not a primer pair with matching annealing temperatures). The nature of the RACE-PCR technique is in the use of one universal primer instead of one sequence specific primer. This eliminates the need for the second sequence-specific primer, but leads to a faster accumulation of the products of non-specific amplification and the faster depletion of the universal primer (the latter might require the use of higher concentrations of the universal primer, and has to be determined experimentally in each particular case). The major advantage of using RACE-PCR in addition to the requirement of the single sequence specific primer) is that the annealing temperatures can be more easily matched by redesigning the universal RACE primers (e.g. modifying their length). Table 1 lists such subsets of universal primers suitable for the RACE-PCR (both Oligo-dT and "URA" primer subsets were used successfully though we prefer the Oligo-dT primers over the "URA" adapter primers, also designed in the course of this work). The designed Oligo-dT primers had predicted Tm's of 68.8°C, 57.1°C and 50.1°C (calculated using on-line software from Sigma-Genosys, Cambridge, United Kingdom). The nearest Tm matched primer was used as the RACE primer for each different degenerate primer to ensure the best performance of RACE-PCR (thus the difference in the annealing temperatures was always kept low). Finally, as a last step of primer design all primer pairs (all universal "URA" primers vs. each individual hybrid partially degenerate primers) were checked for any possible dimer formation. Two nucleotides long overlap at the primers' 3' end was not considered as leading to dimer formation, but three and higher overlaps were disallowed. The primer design is therefore a careful balance between the need to allow for all known (and potentially unknown) degenerate positions and the requirement of limited degeneracy, matched annealing temperatures, absence of secondary structures, no self annealing, no primer dimers and the ease of subsequent cloning. Table 1 lists all the primers designed in our investigation and might serve an example of successful primer design for a similar experiment.

### Optimisation of the amplification conditions

**Figure 4:**
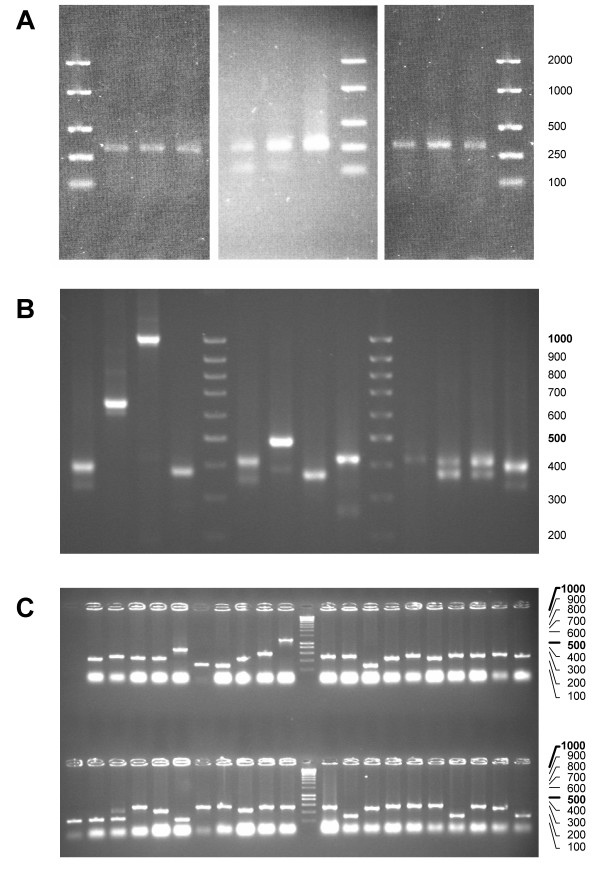
**PCR using the designed primers**. (A) The optimisation of the annealing temperatures. Each hybrid primer was tested for amplification at three different annealing temperatures, roughly chosen as T_m_-7°C, T_m_-5°C and T_m_-3°C (based on T_m _values calculated using on-line primer design software from Sigma-Genosys). Hybrid primer M22F (panel A, left gel) does not show any preference for annealing temperature. Primer M15F (panel A, middle) shows strong preference for T_m_-7°C. Primer 06F (panel A, right panel) exemplifies an apparent preference for T_m_-5°C. Note, although all the above amplifications were performed simultaneously under identical conditions and using gradient PCR machine, no quantitative estimate of the amplification efficiencies can be made. However, relative differences due to the change of the annealing temperature could and have been detected. (B) A gel electrophoresis exemlifying RACE-PCR amplification of *Agelena orientalis *cDNA using hybrid primers (left to right) M31F, M33F, M35F, M37F, M39F, M41F, M43F, M45F, M47F, M49F, M51F, M31F (identical to the leftmost PCR product). Nearly all primers (except for M37F and M47F) yielded more than one group of products. Amplification conditions were optimised for each hybrid primer, as described above (panel A) and the oligo-dT primer with the matching T_m _(see Table 1). (C) High throughput positive clone selection by direct PCR amplification from bacterial colonies. The lower band (just above 100 bp marker) corresponds to amplified plasmid fragments without inserts. The upper bands are the amplified fragments with inserts. Plasmid specific primers PBS-F and PGM-2R were used (see Table 1 for sequences).

The guide on choosing the PCR conditions and various key optimisation procedures can be found elsewhere [18,36-39]. Below we describe key amplification conditions used in our investigation and depict additional optimisation steps, easily applicable to any similar cloning problem. Because of the degeneracy, the primer mixtures have a wide range of Tm values. Since the correct matching sequence is unknown (otherwise no degeneracy would be needed) it is impossible to predict the annealing temperature theoretically. It can only be determined experimentally. Therefore each individual partially degenerate primer was probed at a range of temperatures (using Mastercycler gradient (96-wells) PCR machine from Eppendorf) and, where necessary, with different RACE-PCR universal (oligo-dT) primers having matching temperatures (Table 1). Only those primers which showed any amplification and the corresponding optimal temperature cycling conditions were used in later investigations. The optimisation of the annealing temperature (see Figure 4A for the example) has shown that temperature differences as low as 2°C could have dramatic effects on the RACE-PCR outcome for some of the degenerate primers (much more so than would such a temperature difference affect a standard PCR). We have also routinely changed the *annealing *temperature through the PCR amplification. The annealing step temperature was always gradually *decreased *at a rate of 0.1–0.2°C per cycle as the amplification progressed, in order to reduce the stringency of annealing and allow primers with mismatches to anneal and to continue the amplification. This has allowed us to maintain the high exponential rate of amplification of the correct fragments. Despite the decrease in the concentration of the matching primers towards the end of the amplification, the proportion of the correct amplified sequences increases and thus the probability and the kinetics rate of annealing the mismatched primers also increases, resulting in a stronger amplification albeit with a few mismatched bases very near the fragment ends (within primer sequences). Our temperature ramp (during the annealing steps) is different from what is commonly known as "touch down" or "step down" approaches, which aim to minimise the need to optimise annealing temperature or buffer conditions and circumvent spurious priming during gene amplification [37,39]. In the "touch down" or "step down" applications PCR priming is normally initiated *above *the optimum annealing temperature for the specific primer to promote a greater *discrimination *between the annealing/amplification for the *most stringently *annealed primer/template pair (versus same primers annealed non-specifically to another place on the DNA template). Such an approach would be detrimental if highly degenerate primers are used (as in the described study) due to the artificially strong advantage that would be given to the primers with higher annealing temperatures, which are not necessarily the primers having correct sequences. In our approach we already start with the temperature finely optimised (within just a few degrees) to achieve higher (or any) yield for the PCR product of expected size (where possible). The very small decrease in the annealing temperature in our protocol is to allow the *mismatched *oligonucleotides to continue to prime PCR amplification at later amplification stages.

### High throughout amplification and cloning

Of the 44 primers that passed our selection criteria described above, 40 have been shown to produce products (i.e. resulted in cDNA amplification). Altogether over 60 distinct cDNAs (or groups of cDNAs of similar lengths) were identified from *Agelena orientalis *total RNA by low stringency 3'-RACE-PCR (since many sets of primers have yielded more than one group of cDNA fragments, by their length). Figure 4B gives just a few examples of the first round of RACE amplifications. Large numbers of amplified fragments meant that we had to streamline the cloning and positive clone selection procedures. Identification of positive clones was carried out by PCR directly from colonies. The primers (PBS-F and PGM-2R) were designed based on the vector sequence (Table 1). The clones were picked using sterile tips and transferred onto a fresh new antibiotic plate first, then using the same tip directly dissolved in the mixtures of PCR reactions. The positive clones and sizes were identified by agarose gel electrophoresis. This was a much quicker and more informative method for screening positive clones than applying an IPTG (blue/white) selection procedure [40]. This had the additional advantage of allowing positive clones to be identified within approximately one day and the sizes of cloned inserts to be simultaneously determined. This allowed us to improve the 'hit' rate with over 50% of clones chosen for further analysis being independent sequences. Traditional approaches relying on plasmid purification and restriction mapping were not suitable for our high-throughput cloning approach, since it was possible that the chosen restriction enzymes might cut within the unknown insert sequences. Figure 4C illustrates some of the positive clones with different insert sizes that were identified by PCR reactions directly form bacterial colonies.

### Sequences identification and analysis

**Figure 5:**
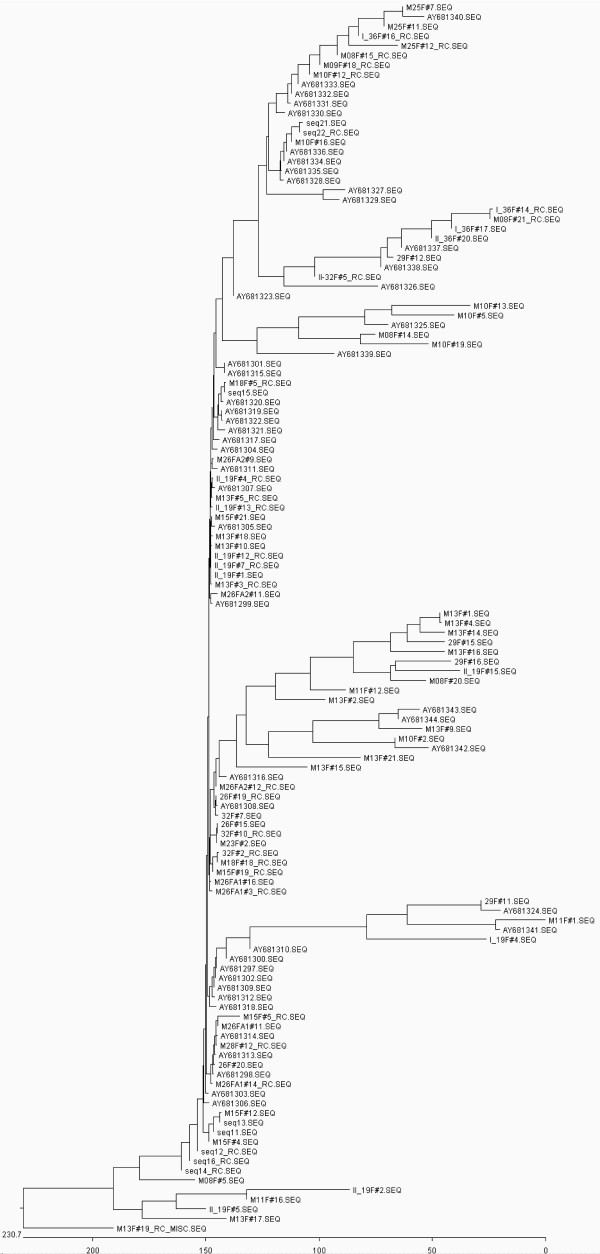
**Philogenetic analysis**. Nucleotide sequence alignment of the 80 novel non-identical sequences obtained by PCR with hybrid primers and *Agelena *sequences (sequence identifiers start with "AY" and correspond to their GenBank entries' names), obtained using EST based approach (as described in [6]). The branch length indicates the extent of sequence similarity.

A total of 226 positive clones with different size inserts were obtained using the RACE-PCR approach described above and confirmed by PCR as positive (i.e. having insert). 130 of these were chosen from different groups of amplified cDNA fragments (preferably having different sizes, see Figure 4C) and were sequenced. Insert sequences generated in this study, plus *Agelena *clones from Kozlov's investigation identified using EST based cloning approach [6], were compared directly against the Genbank database (blastn). It was discovered that there were no significant sequence similarities between *Agelena orientalis *sequences (ours and from Kozlov *et al*. [6]) and published Genbank entries. This was not unexpected since *Agelena orientalis *venom has not previously been studied at the mRNA level. But this has also proved the power of our cloning approach. Out of the 130 non-identical sequences generated by RACE-PCR, 50 sequences matched the sequences form the EST collection reported independently by Kozlov *et al*. [6] with 100% sequence identity. The remaining 80 clones did not fully match reported sequences (sequence identity ranging between 93% and 99% in most cases, with a few sequences nearly having no identifiable sequence similarity to the EST clones) and are likely to represent additional toxins or toxin-like sequences. We believe that this validated our approach to cloning and demonstrated an advantage of using a RACE-PCR approach in that it has proven extremely effective for discovering novel sequences and compares very favourably with the EST based approach (in which only 48 novel sequences were identified from over 2100 raw EST sequences) [6]. The phylogenetic tree obtained following multiple alignment of all of the novel sequences (including the 48 novel cDNA reported by Kozlov *et al*. [6] is shown in Figure 5. This illustrates the extent of the sequence similarities between different *Agelena *sequences. Most of the sequences are rather alike with an average of 90% sequence similarity, whilst some sequences show larger differences, such as I_19F#4, II_19F#2, II_19F#5, II_19F#15, 29F#15, 29F#16, M13F#1, M13F#2, M13F#4, M13F#9, M13F#14, M13F#15, M13F#16, M13F#17, M13F#19, M13F#21, M08F#5, M08F#14, M08F#20, M10F#5, M10F#13, M10F#19, M11F#1, M11F#12, M11F#16 (see Figure 5). These possess only short regions of high sequence homology, with the rest of the sequences having no clearly identifiable similarities. Some clones (e.g. 32F#7, M25F#12, M09F#18) might represent novel splice variants of previously reported sequences. Additional material available on-line [see Additional file 1] contains all the sequences reported in Figure 5 and summarises the relationships of our sequences with the previously reported *Agelena *sequences.

## Conclusion

The RACE RT-PCR strategy is a useful technique for amplifying specific regions of mRNA between a defined and known internal site sequence and an unknown sequence located at either the 3' or 5' end. When RACE-RT PCR is combined with the application of degenerate primers the method becomes a very effective way to clone and identify novel cDNA fragments. Previously, such an approach has been used successfully to clone and sequence cDNAs encoding insecticidal peptides from primitive hunting spiders [41]. The application of PCR-based cloning directly complements an EST-based approach [42]. However, PCR-based cloning proffers a distinct advantage whereby even rare transcripts can be identified, i.e. low-abundance transcripts are similarly well represented following amplification and are not lost in preference to higher abundance molecules. This contrasts with EST cloning which can often result in multiple copies of the same abundant mRNA/cDNAs being cloned, unless normalised libraries are exploited [43]. Another important issue is the proportional representation of individual sequences at the outcome of the experiment. EST based strategies (unless used with normalised libraries) preserve the information on relative abundance of respective cDNAs, whilst a PCR based approach might not, unless it is a truly quantitative qRT-PCR, see e.g. [44]. The latter is especially valid if no truly universal oligonucleotide primers (capable of simultaneously amplifying the whole family of mRNAs/cDNAs under investigation) are used. The case presented here is a good example of such an approach. We have not preserved any quantitative information on toxin mRNA/cDNA expression, but compared to the EST approach reported in [6] we were able to identify more than twofold the number of different sequences from the same material. Our oligonucleotide selection algorithm is therefore superior if the discovery of new genes is sought, but should not be used if quantitative analysis of mRNA/cDNA expression is required.

As interest in the field of neuro-modulatory molecules continues to expand, our approach contributes significantly through the provision of an effective high-throughput methodology to identify novel neurotoxin-like peptide sequences from the venom of *Agelena orientalis *species of spiders. The collective use of published neuro-toxin sequence information, sets of specifically designed partially degenerate hybrid primers and streamlined cloning and positive clone selection procedures have enabled us to identify a large set (~130) of toxin-like sequences from *Agelena orientalis*. This is an invaluable resource although in all probably it represents only a very small fraction of the overall number of functional spider toxin peptides that are still awaiting discovery. The sequences identified with RACE-PCR include 80 putative novel toxin-like sequences, in addition to the ones matching the sequences obtained independently by an EST-based approach [6]. These sequences are indicative of the vast repertoire and/or diversity of toxins, or toxin-like proteins, present in *Agelena orientalis *venom. These spider venom toxins are a rich biological resource of active components and this presents an opportunity to develop further biological tools for neuroscience, in drug target discovery and possibly as effective bio-pesticides. With upwards of 100,000 species of spider thought to exist, and each showing a propensity for producing diverse peptide pools, many more neurotoxin peptides await discovery. It is likely that a high percentage of these could be exploited in a wide range of novel applications, and profit both research and commercial industries.

## Methods

The method development and optimisation procedures are described earlier in this paper. Other methods are as follows: the Aurum total RNA mini kit from BIO-RAD was used for total RNA purification. The spider RNA was purified according to manufacturer's recommendations using Spider glands (kindly provided by E.V.Grishin, Shemyakin- and Ovchinnikov Institute of Bioorganic Chemistry, Russian Academy of Sciences, Moscow). cDNAs were synthesised by using RevertAid™ M-MuLV Reverse Transcriptase and "OLIGO-dT-21" or "URA_L" DNA primers (see Table 1) according to manufacturer recommendations. cDNA was synthesised for 30 min at 42°C, followed by 30 min at 50°C. The reaction was stopped by heating the mixture at 70°C for 10 minutes and chilling on ice. cDNA was aliquoted and stored frozen at -20°C (cDNA was further diluted ×50 fold before use in PCR). RACE-PCR conditions were outlined above. Briefly, an incubation at 95°C for 2 min (hot start) was followed by 15–30 cycles (typically 25 cycles) of 95°C for 1 min, T_opt _(the optimised annealing temperature) for 1 min (and 0.1 – 0.2 °C drop per cycle), 72°C for 1 min; The reaction was then kept at 72°C for 5 min and then held at 4°C. Red Taq polymerase (Bioline) or Pfx (Invitrogen) were used. To improve reproducibility of the PCR amplifications, all reagents except the sequence-specific primers (but including polymerase) were mixed to make a master solution and transferred into individual PCR tubes on ice. Then the sequence-specific primers were added (also on ice) and the tubes were transferred into a preheated PCR block. "HGAP-F62" and "HGAP-R62" GAPDH primers (Table 1) were used to amplify mouse cDNA in optimisation experiments, mouse cDNA amplification was also used as a positive control throughout. All oligonucleotide primers were custom-made at SIGMA-Genosys (Cambridge, United Kingdom). Competent cells (*E.coli *strain cMAX5α, BIO-RAD) for chemical transformation were prepared as follows. 100ul cells (from -20°C stock) were added into 5 ml LB media and cultured overnight at 37°C. 10ul cells from the overnight culture were added to 5 ml LB containing 20 mM Mg^2+ ^media and grown at 28°C until OD_550_~0.5. Cells were then transferred into 50 ml Falcon tubes, chilled on ice for 15 minutes and centrifuged at 1000 g for 15 min. The cell pellet was resuspended in 17 ml of the FSB buffer (1/3 of the original culture volume) and kept on ice for 15 minutes. The centrifugation step was repeated once, after which the cells were resuspended in 4 ml FSB buffer (to 1/12.5 of the original culture volume). 140 ul DMSO was added to the cell suspension (3.5% final concentration) and kept on ice for 5 minutes. Another aliquot of 140ul DMSO was added (to make 7% final DMSO concentration) and the cells were kept on ice for a further 15 minutes before being aliquoted into chilled tubes and flash frozen in dry ice. Transformation efficiency of the competent cells was ca. 10^8 ^transformants/μg DNA (heat shock was at 42°C/40 seconds). The use of cells with high transformation efficiency has proven highly beneficial especially when cloning low abundance PCR products. The T-Easy Vector (Promega) was used to clone Taq amplified sequences (both with and without PCR product purification) and according to the manufacturer recommended procedure (ligation was at 25°C/1 hour). Plasmid purification was using Promega Miniprep Kit. Sequencing was done commercially by Marcogen (Korea).

## Abbreviations

*RT-PCR*, reverse transcriptase polymerase chain reaction; *RACE-PCR*, rapid amplification of cDNA ends polymerase chain reaction; *EST*, expressed sequence tag; *RLM-RACE-PCR, RNA *ligase-mediated rapid amplification of cDNA ends polymerase chain reaction; *LS-SSP-PCR*, low stringency single specific primer polymerase chain reaction; *AP-PCR*, arbitrarily primed polymerase chain reaction; *DD-PCR*, differential display polymerase chain reaction; *AFLP*, amplified fragment length polymorphism; *CODEHOP*, COnsensus-DEgenerate Hybrid Oligonucleotide Primers.

## Authors' contributions

ZP carried out bulk of PCR amplifications, participated in the sequence alignments and statistical analysis. RB participated in the design of PaBaLiS oligonucleotides, carried out trial experiments and drafted the manuscript. AL mastered RNA extraction from the glands of *Agelena orientalis *species of spider and cDNA synthesis and also participated in the sequence alignments. MS conceived of the study, devised the PaBaLiS approach to primer design, constructed the bulk of PaBaLiS oligonucleotides and coordinated the study. All authors read and approved the final manuscript.

## Supplementary Material

Additional file 1 Novel sequences identified using PaBaLiS approach and *Agelena orientalis *cDNA.. The Table contains all the sequences reported in this manuscript (naming convention is the same as in Figure 5 of this manuscript) and summarises the relationships of our sequences with the previously reported *Agelena orientalis *sequences. Click here for file

## Figures and Tables

**Table 1:** PaBaLiS oligonucleotide primers designed in this study.

Primer name	Primer sequence
01F	GWNTGYGAYGGIAAYGA
06F	TGYATTGCIRARGAYTAYGG
08F	AAYTGYAAYTGYTGYGG
10F	GATGARAAYTGYTGYAARAA
12F	GGAATTCARGCIGARCCNAA
14F	CCACAYACNTGYTGYTGGGG
15F	AARGAYTGYTGYCARTG
17F	CAYGGATGYTGYTGGCCNTG
19F	GAYTGYGAYTGYTGYGG
22F	TAYATGTTYGGNGGNTG
26F	GAAGAATYWAARGGNGARGA
29F	AARTGYTGYGAYGAYTG
32F	GTCGARTGYTGYAAYGARTG
36F	GATTGGTAYGAYGARTGYTG
M08F	CCAGGAAAIRAYSARTGYTG
M09F	TAYYTTTTYGGIGGNTG
M10F	TGYCARTGYGTNATGAA
M11F	ATGTGYGARTGYAAYATHATGGG
M12F	GGAGRNGAYTGYGAYGG
M13F	ACNTGYGAYTGYTGYGG
M14F	GAYTGYSARTGYTGYGG
M15f	TGGATHGARGAYGGNTGGAA
M16F	TGYTGYGGNGARATGTGGTG
M17f	ATGTAYTGYMGNATGAT
M18f	TGYTGYGARGGIYTITAYTG
M19f	TGYTGYGAYGGITGGMGITG
M20f	TGYTGYGAYGGITAYTAYTG
M22f	TGYTGYWSIGGITAYTAYTG
M23f	TGYTGYGAYGGITTYTAYTG
M24f	TGYTGYGARGGITTYTAYTG
M25f	TGYTGYAARGGITAYTAYT
M26f	TGYTGYTCAGGNTTTAGATG
M28f	TGYTGYCARGGITAYTAYTG
M31F	CTTATTTYKGCHATKGTCTT
M33F	ATAACAAAATAACCATGAGAACTA
M35F	GAATWTTACAAAWTRACMATGA
M37F	TGAGAWAMGAAWYACCACAAA
M39F	AAGACAAAGGATTATAAGATG
M41F	TTGAAACAGAAAGAGAAGAATG
M43F	ATGAAGCTTCTTTTAGCAA
M45F	ATGARARCTATMATTTYYYT
M47F	AAATTCAGGAGTCGTACAATG
M49F	TCAGGTATCCAAGAACAATG
M51F	TCCTGGATACAAACCTAAA

Non standard and degenerate nucleotide positions are defined as follows: R = A+G, Y = C+T, N = A+C+G+T, M = A+C, W = A+T, D = G+A+T, I = Inosine, K = G+T, H = A+T+C, S = G+C, B = G+T+C, V = A+C+G

**Table 2:** Oligonucleotide primers designed for RACE-PCR and for the selection of positive clones

Primer name	Primer sequence
URA_L*	CTGCAGATCTAGAAGCTTTTTTTTTTTTTT
URA_N	GGAATTCTGCAGATCTAGAAG
URA_T	GGAATTCTGCAGATCTA
	
OLIGO-dT-21**	TTT TTT TTT TTT TTT TTT TTT
OLIGO-dT-24	TTT TTT TTT TTT TTT TTT TTT TTT
OLIGO-dT-27	TTT TTT TTT TTT TTT TTT TTT TTT TTT
	
hGAP-F62***	AGCCAAAAGGGTCATCATCT
hGAP-R62	GCCTGCTTCACCACCTTC
	
pBS-F****	GGTTTTCCCAGTCACGA
pGM-2R	ATGCTTCCGGCTCGTATGT

* URA (all primers) – RACE-PCR primers;

** OLIGO-dT (all primers) are for RACE-PCR, OLIGO-dT-21 was also used for cDNAs synthesis;

*** hGAP (both primers) – for positive controls (with mouse cDNA);

**** pBS-F and pGM-2R designed for the selection of positive clones from bacterial colonies.
