# Supplementary material for: Selection strategy and the design of hybrid oligonucleotide primers for RACE-PCR: cloning a family of toxin-like sequences from Agelena orientalis
Source: BMC Mol Biol. 2007 May 11;8:32. doi: 10.1186/1471-2199-8-32 (PMC1876241; doi:10.1186/1471-2199-8-32)
Supplement: Additional file 1 — Novel sequences identified using PaBaLiS approach and Agelena orientalis cDNA.. The Table contains all the sequences reported in this manuscript (naming convention is the same as in Figure 5 of this manuscript) and summarises the relationships of our sequences with the previously reported Agelena orientalis sequences. [file 1471-2199-8-32-S1.pdf]

Pan et al. sequences		Best matching Agelena database entry	
NAME	SEQUENCE	Accession number	Name
M26FA1#14	AAAATTGCATTATATTTTATTGAAATGATCAGAAATACAGAATTTCATAAT<br>ATGAATTAACATATATAGAATTTTGACATATGCTCAACTTTGCTATTTCAA<br>TACAAATGTTGGTAACAAATTGAAATCAAAGATTTTTTCCAACCGTCTTC<br>TATCCAAGACATCTGCTATCCCAAATGCTTAATTCCTTA	AY681298	Agel_01
26F#20	CAAGGAATTAAGCATTTGGGATAGCAGATGTCTTGGATAGAAGACGGTT<br>GGAAAAAATCTTTGATTTCAATTTGTTACCAACATTTGTATTGAAATAGC<br>AAAGTTGAGCATATGTCAAAATTCTATATATGTTAATTCATATTATGAAAT<br>TCTGTATTTCTTATTGATTCAATAAAATATAATGCAATTTT	AY681298	Agel_01
M26FA2#11	CCAGGAATTAAGCATTTGGGATAGCAGATGTCTTGGATAGAAGACGGA<br>TGGAAAAAATCTTTCATTTCAATTTGTTACCAACATTTCAATTGAAATATC<br>AAACTGAAGCATATATCAAAATTCTATATATGTTAATTCATATTATGAAAT<br>TCTGTATTTCAGATCGTTTCAATAAAATATAATGCAAATTT	AY681299	Agel_02
32F#2	AAAATTGTATTATATTTTATTGAAATGATCAGAAATACAGAATTTCATATT<br>ATGAATTAACATATATAGAATTTTGATATATGCTTCAGTTTGATATTTCAA<br>TTCAAATGTTGGTAACAAATTGAAATCAAAGATTTTTTCCATCCATCTTC<br>TATCCAAGACATCTGCTGTCCCAAATGCTTAATTCCTTGCATTTTAATCC<br>TGAGCAACACCTTGGAC	AY681299	Agel_02
32F#10	AAAATTTCATTATATTTTATTGAAATGATCAGAAATACAGAATTTCATAAT<br>ATGAATTAACATATATAGAATTTTGATATATGCTCCAGTTTGATATTTCTT<br>TTCAAATATTGGTAACAAATTGAAATCAAAGATTTTTTCCATCCGTCTTC<br>TATCCAAGACATCTGCTATCCCAAATACTTAATTCCTTGCATTTTAATCC<br>TGAGCAGCACCTTGGAC	AY681299	Agel_02
M15F#4	TTGGATAGAGGACGGCTGGAAAAAATCTTTGATTTCAATTTGTTACCAA<br>CATTGAATTGAAATATCACACTGGAGCATATGTCAAAATTCTATATATGT<br>TAATTCATATTATGAAATTCTGTATTTCTTATCGATTCAATAAAATATAAT<br>GCAATTTT	AY681303	Agel_06
II_19F#12	AAAATTGCATTATATTTTATTGAAATGATCAGAAATACAGAATTTCGTAAT<br>ATGAATTAACATATATAGAATTTTGATATATGCTCCAGTTTGATATTTCAA<br>TTGAAATGTTGGTAACAAATTGAAATCAAAGATTTTTTTCCAACCGTCTT<br>CTATCCAAGACATCTGCTATCCCAAATGCTTAATTCCTTGCATTTTAATC<br>CTGAGCAGCACCTTGGACCACTCAATGCATTACAAAATCTATTATGAGG<br>AAGACATCCCCTTTCACCTTCAAAAAGCTGTAAACCTTCTTCTACGGGT<br>ACAGCTTCAATCATGGAGAAGACCATTGCAGAAATAAGTAAAAGGGAAA<br>TTATAGCTTTCATTGTTATTTTATTATAATCCTTTTGTCTC	AY681307	Agel_10
II_19F#13	AAAATTGCATTATATTTTATTGAAATGATCAGAAATACAGAATTTCGTAAT<br>ATGAATTAACATATATAGAATTTTGATATATGCTCCAGTTTGATATTTCAA<br>TCCAAATGTTGGTAACAAATTGAAATCAAAGATTTTTTTCCAACCGTCTT<br>CTACCCAAGACATCTGCTATCCCAAATGCTTAATTCCTTGCATTTTAATC<br>CTGAGCAGCACCTTGGACCACTCAATGCATTACAAAATCTATTATGAGG<br>AAGACATCCCCTTTCACCTTCAAAAAGCTGTAAACCTTCTTCTACGGGT<br>ACAGCTTCAATCATGGAGAAGACCATTGCAGAAATAAGTAAAAGGGAAA<br>TTATAGCTTTCATTGTTATTTTATTATAATCCTTTTGTCTC	AY681307	Agel_10
II_19F#1	GAGACAAAAGGATTATAATAAAATAACAATGAAAGCTATAATTTCCCTTT<br>TACTTATTTCTGCAATGGTCTTCTCCATGATTGAAGCTGTACCCGTAGA<br>AGAAGGTTTACAGCTTTTTGAAGGTGAAAGGGGATGTCTTCCTCATAAT<br>AGATTTTGTAATGCATTGAGTGGTCCAAGGTGCTGCTCAGGATTAAAAT<br>GCAAGGAATTAAGCATTTGGGATAGCAGATGTCTTGGATAGAAGACGG<br>TTGGAAAAAAATCTTTGATTTCAATTTGTTACCAACATTTCAATTGAAATA<br>TCAAACTGGAGCATATATCAAAATTCTATATATGTTAATTCATATTACGA<br>AATTCTGTATTTCTGATCATTTCAATAAAATATAATGCAATTTT	AY681307	Agel_10

II_19F#4	AAAATTGCATTATGTTTTATTGAAATGATCAGAAATACAGAATTTCGTAA<br>TATGAATTAACATATATAGAATTTTGATATATGCTCCAGTTTGATATTTCA<br>ATCCAAATGTTGGTAACAAATTGAAATCAAAGATTTTTTTCCAACCGTCT<br>TCTATCCAAGACATCTGCTATCCCAAATGCTTAATTCCTTGCATTTTAAT<br>CCTGAGCAGCACCTTGGACCACTCAATGCATTACAAAATCTATTATGAG<br>GAAGACATCCCCTTTCACCTTCAAAAAGCTGTAAACCTTCTTCTACGGG<br>TACAGCTTCAATCACGGAGAAGACCATTGCAGAAATAAGTAAAAGGGAA<br>ATTATAGCTTTCATTGTTATTTTATTATAATCCTTTTGTCTC	AY681307	Agel_10
II_19F#7	AAAATTGCATTATATTTTATTGAAATGATCAGAAATACAGAATTTCGTAAT<br>ATGAATTAACATATATAGAATTTTGATATATGCTCCAGTTTGATATTTCAA<br>TTGAAATGTTGGTAACAAATTGAAATCAAAGATTTTTTTCCAACCGTCTT<br>CTATCCAAGACATCTGCTATCCCAAATGCTTAATTCCTTGCATTTTAATC<br>CTGAGCAGCACCTTGGACCACTCAATGCATTACAAAATCTATTATGAGG<br>AAGACATCCCCTTTCACCTTCAAAAAGCTGTAAACCTTCTTCTACGGGT<br>ACAGCTTCAATCATGGAGAAGACCATTGCAGAAATAAGTAAAAGGGAAA<br>TTATAGCTTTCATTGTTATTTTATTATAATCCTTTTGTCTC	AY681307	Agel_10
M13F#3	AAAATTGCATTATATTTTATTGAAATGATCAGAAATACAGAATTTCGTAAT<br>ATGAATTAACATATATAGAATTTTGATATATGCTCCAGTTTGATATTTCAA<br>TTGAAATGTTGGTAACAAATTGAAATCAAAGATTTTTTTCCAACCGTCTT<br>CTATCCAAGACATCTGCTATCCCAAATGCTTAATTCCTTGCATTTTAATC<br>CTGAGCAGCACCTTGGACCACTCAATGCATTACAAAATCTATTATGAGG<br>AAGACATCCCCTTTCACCTTCAAAAAGCTGTAAACCTTCTTCTACGGGT<br>ACAGCTTCAATCATGGAGAAGACCATTGCAGAAATAAGTAAAAGGGAAA<br>TTACAGCTTTCATTGTTATTTTATTATAATCCTTTTGTCTC	AY681307	Agel_10
M13F#5	AAAATTGCATTATATTTTATTGAAATGATCAGAAATACAGAATTTCGTAAT<br>ATGAATTAACATATATAGAATTTTGATATATGCTCCAGTTTGATATTTCAA<br>TCCAAATGTTGGTAACAAATTGAAATCAAAGATTTTTTTCCAACCGTCTT<br>CTATCCAAGACATCTGCTATCCCAAATGCTTAATTCCTTGCATTTTAATC<br>CTGAGCAGCACCTTGGACCACTCAATGCATTACAAAATCTATTATGAGG<br>AAGACATCCCCTTTCACCTTCAAAAAGCTGTAAACCTTCTTCTACGGGT<br>ACAGCTTCAATCACGGAGAAGACCATTGCAGAAATAAGTAACAGGGAA<br>ATTATAGCTTTCATTGTTATTTTATTATAATCCTTTTGTCTC	AY681307	Agel_10
M13F#10	GAGACAAAAGGATTATAATAAAATAACAATGAAAGCTATAATTTCCCTTT<br>TACTTATTTCTGCAATGGTCTTCTCCATGATTGAAGCTGTACCCGTAGA<br>AGAAGGTTTACAGCTTTTTGAAGGTGAAAGGGGATGTCTTCCTCATAAT<br>AGATTTTGTAATGCATTGAGTGGCCCAAGGTGCTGCTCAGGATTAAAAT<br>GCAAGGAATTAAGCATTTGGGATAGCAGATGTCTTGGATAGAAGACGG<br>TTGGAAAAAAATCTTTGATTTCAATTTGTTACCAACATTTCAATTGAAATA<br>TCAAACTGGAGCATATATCAAAATTCTATATATGTTAATTCATATTACGA<br>AATTCTGTATTTCTGATCATTTCAATAAAATATAATGCAATTTT	AY681307	Agel_10
M13F#18	GAGACAAAAGGATTATAATAAAATAACAATGAAAGCTATAATTTCCCTGT<br>TACTTATTTCTGCAATGGTCTTCTCCATGATTGAAGCTGTACCCGTAGA<br>AGAAGGTTTACAGCTTTTTGAAGGTGAAAGGGGATGTCTTCCTCATAAT<br>AGATTTTGTAATGCATTGAGTGGTCCAAGGTGCTGCTCAGGATTAAAAT<br>GCAAGGAATTAAGCGTTTGGGATAGCAGATGTCTTGGATAGAAGACGG<br>TTGGAAAAAAATCTTTGATTTCAATTTGTTACCAACATTTCAATTGAAATA<br>TCAAACTGGAGCATATATCAAAATTCTATATATGTTAATTCATATTACGA<br>AATTCTGTATTTCTGATCATTTCAATAAAATATAATGCAATTTT	AY681307	Agel_10
M26FA2#12	AAAATTGCATTATATTTTATTGAAATGATCAGAAATACAGAATTTCATAAT<br>ATGAATTAAAATATACAGAATTTTGATATATGCTGCAGTTGGATATTTCA<br>ATTCAAATGTTGGTAACAAATTGAAATCAAAGATTTTTTTCCAACCGTTT<br>TCTATCCAAGACATCTGCTATCCCAAATGCTTAATTCCTTG	AY681308	Agel_11
26F#19	AAAATTGCGTTATATTTTATTGAAATGATCAGAAATACAGAATTTCATAAT<br>ATGAATTAAAATATACAGAATTTTGATATATGCTGCAGTTGGATATTTCA<br>ATTCAAATGTTGGTAACAAATTGAAATCAAAGATTTTTTTCCAACCGTCT<br>TCTATCCAAGACATCTGCTATCCCAAATGCTTAATTCCTTG	AY681308	Agel_11

32F#7	GTCCAAGGTGCTGCTCAGGATTAAGATGCAAGGAATTAAGCATTTGGG<br>ATAGCAGATGTCTTGGATAGAGGACGGTTGGAAACAGCATATATCAAAA<br>TTCTGTATATTTTAATTCATATTATGAAATTCTGTATCTCTGATCATTTCA<br>ATAAAATATAATGCAATTTT	AY681308	Agel_11
M15F#12	TGGAAAAAATCTTTGATTTCAATTTGTTACCAACATTGGTATTGAAATAG<br>CAGAGTTGAGCATATGTCCAAATTCTATATATGTTAATTCATATTATGAA<br>ATTCTGTATTTCTTATCCATTCTAGAAAATATAATGCAATT	AY681310	Agel_13
M28F#12	AAAATTGCATTACATTTTATTGAATCGATAAGAAATACAGAATTTCATAAT<br>ATGAATTAATATATACAGAATTTTGACATATGCTCAACTTTGCTATTTCAA<br>TACAAATGTTGGTAACAAATTGAAATCAAAGATTTTTTCCAACCGTCTTC<br>TATCCAAGACATCTGCTATCCCAAATGCTTAATTCCTTG	AY681313	Agel_16
M26FA1#11	CAAGGAATTAAGCATTTGGGATAGCAGATGTCTTGGATAGAAGACGGTT<br>GGAAAAAATCTTTGATTTCAATTTGTTACCAACATTTGTATTGAAATAGC<br>AAAGTTGAGCATATGTCAAAATTCTATATATGTTAATTCATATTATGAAAT<br>TCTGTATTTCTTATCGATTCAATAAAATGTAATGCAATTTT	AY681313	Agel_16
M15F#5	AAAAAGGCATTACATTTTATTGAATCGAAAAGAAATACAGAATTTCATAA<br>TATGAATTAACATATATAGAATTTTGACATATGCCCAACTTTGCTATTTCA<br>AAACAAAGGGGGGGAACAAATTGAAATCAAAGATTTTTTCCCCCCCTCC<br>CCAATCCAA	AY681313	Agel_16
SEQ14	CCCAAATAGTTAATTCCTTGCATCTTAATCCTGAGCAGCACCTTGGACC<br>ACTCAATGCGTTACAAAATCTATTATGAGGAAGACATCCTCCCCTTTCAT<br>CTTCAGAAAGCTGTAAACCTTCTTCTTCAGGTACAGCTGCAATCATGGA<br>GAAGACAATGGCCAAAATAAG	AY681313	Agel_16
SEQ16	CCCAAATAGTTAATTCCTTGCATCTTAATCCTGAGCAGCACCTTGGACC<br>ACTCAATGCGTTACAAAATCTATTATGAGGAAGACATCCTCCCCTTTCAT<br>CTTCAGAAAGCTGTAAACCTTCTTCTTCAGGTACAGCTGCAATCATGGA<br>GAAGACAATAGCAAAAATAAG	AY681313	Agel_16
SEQ12	CCCAAATGGTTAATTCCTTGCATTTTAATCCTGAGCAGCACCTTGGACC<br>ACTCAATGCGTTACAAAATCTATTATGAGGAAGACATCCTCCCCTTTCAT<br>CTTCAGAAAGCTGTAAACCTTCTTCTTCAGGTACAGCTGCAATCATGGA<br>GAAGACAATTGCCGAAATAA	AY681318	Agel_21
M18F#5	AAAACTACATTATATTTTATTGAAATGATATGATTAGAAATACAGAATTTC<br>ATAATATGAATTAACATGTATAGAATTTTGATATATGCTCCAGTTTGATAT<br>TTCAATTTAAATGTTGGTAACAAATTGAAATCAAAGATTTTTTACAACCG<br>TCTTAAAGACATTTGCTAGCCCAAATGTTTAATTCCTT	AY681320	Agel_23
SEQ13	CTTATTTTGGCCATTGTCTTCTCCATGATTGAAGCTGTACCCTTAGAAGA<br>AGGTTTACAGCTTTTTGAAGGTGAAAGGGGATGTCTTCCTAGAAATAAA<br>TTTTGTAATCCATCAAGTGGACCAAGGTGCTGCTCAGGATTAACATGCA<br>AGGAATTAACCATTT	AY681321	Agel_24
SEQ11	GTCTTCTCCATGATTGAAGCTGTACCCGTAGAAGAAGGTTTACAGCTTT<br>TTGAAGGTGAAAGGGGAGGATGTCTTCCTAGAAATAAATTTTGTAATCC<br>ATCGAGTGGACCAAGGTGCTGCTCAGGATTAACATGCAAGGAATTAAC<br>CATTT	AY681322	Agel_25
SEQ15	CTTATTTCGGCAATTGTCTTCTCCATGATTGAAGCTGTACCCGTAGAAG<br>AAGGTTTACAGCTTTTTGAAGGTGAAAGGGGAGGATGTCTTCCTAGAAA<br>TAAATTTTGTAATCCATCGAGTGGACCAAGGTGCTGCTCAGGATTAACA<br>TGCAAGGAATTAAACATTT	AY681322	Agel_25
M18F#18	AAATTGTATTATATTTTATTGAAATGATCAGAAATACAGAATTTCATATTA<br>TGAATTAACATATATAGAATTTTGATATATGCTTCAGTTTGATATTTCAAT<br>TCAAATGTTGGTAACAAATTGAAATCAAAGATTTTTTCCATCCATCTTCT<br>ATCCAAGACATCTGCTGTCCCAAATGCTTAATTCCTT	AY681323	AgorTX_A3
M23F#2	CAAGGAATTAAGTATTTGGGATAGCAGATGTCTTGGATAGAAGACGGAT<br>GGAAAAAATCTTTGATTTCAATTTGTTACCAATATTTGAAAAGAAATATC<br>AAACTGGAGCATATATCAAAATTCTATATATGTTAATTCATATTATGAAAT<br>TCTGTATTTCTGATCATTTCAATAAAATATAATGAAATTTT	AY681323	AgorTX_A3

M26FA1#16	CAAGGAATTAAGCATTTGGGATAGCAGATGTCTTGGATAGAAGACGGTT<br>GGAAAAAATCTTTGATTTCAATTTGTTACCAACATTTGAATTGAAATATC<br>AAACTGGAGCATATATCAAAATTCTATATATGTTAATTTATATTATGAAAT<br>TCTGTATTTCTGATCATTTCAATAAAATATAATGCAATTT	AY681323	AgorTX_A3
M26FA1#3	AAAAAAATTGCATTATATTTTATTGAAATGATCTGAAATACAGAATTTCAT<br>AATATGAATTAACATATATAGAATTCTGATATATGCTCCAGTTTGATATTT<br>CAATTCAAATGTTGGTAACAAATTGAAATCAAAGATTTTTTCCAACCGTC<br>TTCTATCCAAGACATCTGCTATCCCAAATGCTTAATTCCTTG	AY681323	AgorTX_A3
M26FA2#9	CAAGGAATTAAGCATTTGGGATAGCAGATGTCTTGGATAGAAGACGGTT<br>GAAAAAAATCTTTGATTTCAATTTGTTACCAACATTTGAATTGAAATATCA<br>AACTGGAGCATATATCAAAATTTTATATATGTTAATTCATATTACGAAATT<br>CTGTATTTCTGATCATTTCAATAAAATATAATGCAATTTT	AY681323	AgorTX_A3
26F#15	CAAGGAATTAAGTATTTGGGATAGCAGATGTCTTGGATAGAAGACGGAT<br>GGAAAAAATCTTTGATTTCAATTTGTTACCAATATTTGAAAAGAAATATC<br>AAACTGGAGCATATATCAAAATTCTATATATGTTAATTCATATTATGAAAT<br>TCTGTATTTCTGATCATTTCAATAAAATATAATGAAATTTT	AY681323	AgorTX_A3
M15F#19	AAAATTGTATTATATTTTATTGAAATGATCAGAAATACAGAATTTCATAAT<br>ATGAATTAGCATATATAGAATTTTGATATATGCTCCAGTTTGATATTTCAA<br>TTCAAATGTTGGTAACAAATTGAAATCAAAGATTTT	AY681323	AgorTX_A3
M15F#21	AAAATCTTTGATTTCAATTTGTTACCAACATTTCAATTGAAATATCAAACT<br>GGAGCATATATCAAAATTCTATATATGTTAATTCATATTACGAAATTCTGT<br>ATTTCTGATCATTTCAATAAAATATAATGCAATTTT	AY681323	AgorTX_A3
29F#11	GAGATGTCGCTACACAGGGAGAGCTGTTCCGTTTATGTGCGTACCTTA<br>ACAACTTCGTTGAGCATGATTCCGGAAACCTAAGCACTTAATTTCTTGC<br>CTATAGTATCATTTTAAAATAAAAAAATTCGATGCAC	AY681324	AgorTX_A4
M25F#11	GCACTTGTCGATACTTCCCAAAATGCATATGTGCTAACGACAATGGGAA<br>ATAAGCCTCGATTTCAATTTGTTACTGCTATTTGAAATGTAATATCAAAC<br>TGGAGCATATATTAAAATTTGTTAATGC	AY681330	mu-2Aaga_06
I_36F#16	ATGCATTAACAAATTTTAATATATGCTCTAGTTTGATATTACATTTCAAAT<br>AGCAGTAACAAATTGAAATCGAGGCTTATCTCCCATTGTCGTTAACACA<br>TATGCATTTTGGGAAGTATCGACAAGTGCAATAATATCCTTTG	AY681332	mu-2Aaga_08
M25F#7	GCACTTGTCGATACTTCCCAAAATGCATATGCGTTAACGACAATGGGAA<br>ATAAGCCTCGATTTCAATTTGTTACTGCTATTTGAAATGTAATATCAAAC<br>TAGAGCATATACTAAAATTTGGTAATGCATAGC	AY681332	mu-2Aaga_08
M08F#15	GCCATGCATTAACAAATTTTAATATATGCTCTAGTTTGATATTACATTTCA<br>AATAGCAGTAACAAATTGAAATCGAGGCTTATTTCCCATTGTCGTTAACA<br>CATATGCATTTTGGGAAGTATCGACAAGTGCAATAATATCCTTTGCAAC<br>AGTATGGACCACTCCAATCTG	AY681332	mu-2Aaga_08
M09F#18	AAAATTGCATTATAATTTATTAAAATGAGCATAAATAAAACATTCCATGCT<br>ATGCATTAACAAATTTTAATATATGCTCTAGTTTGATATTACATTTCAAAT<br>AGCAGTAACAAATTGAAATCGAGGCTTATTTCCCATTGTCGTTAACACA<br>TATGCATTTTGGGAAGTATCGACAAGTGCAATAATATCCTTTGCAGCAG<br>TATGGACCACTCCAATCTGCACATTGTTGACTTTCACCAACG	AY681332	mu-2Aaga_08
M10F#12	GCTATGCATTAACAAATTTTAATATATGCTCCAGTTTGATATTACATTTCA<br>AATAGCAGTAACAAATTGAAATCGAGGCTTATTTCCCATTGTCGTTAACA<br>CATATGCATTTTGGGAAGTATCGACAAGTGCAATAATATCCTTTGCAGC<br>AGTATGGACCACTCCAATCTGCACATTGTTGACTTTCACCAACGCAGTC<br>CCCCCTTTCCCCTTCAAATATCTTTAAACCTTCTTCTAAGGATACAGC	AY681332	mu-2Aaga_08
M25F#12	ATGCTTTAGCAAAGTTTAAAAAAAGCTCCAGTTTGGCACCACTTTTCAAA<br>TACCTTTCACAAATTGAAATCCAGGCTTATTTCCCGTTGTACTTTCCACT<br>TATGCTTTTCGGGAAGTATTTGCGAGATGC	AY681333	mu-2Aaga_09

SEQ22	TTCCTGTTTGATANTGCATTTTAAATGTTGGTAATAATATGAAATTGAAG ATTACTTTCCACTGTCTTTCCTACATATGCATTTTGGAAAGTATCGACAA GTGCAATAATATCCTGAGCAGCAGTGGGGACCAGCCCAATCTGCACAC TGTTGATTTTCACCAACGCATCCCCTCTCTCCTTCAAAGAGCTGTAAAC CTTCTTCCAAGGAAATCGCTTCAATCACGGCAAAGACCATAGCAGAAAG AAGTAACAGAGAAATGATAGTTCTCATGGTTATTTTGTTAT	AY681335	mu-2Aaga_11
M10F#16	CAAAAGGGTTATAACAAAATAACCATGAGAACTATCATTTCTCTGTTACT TCTTTCTGCTATGGTCTTCGCCGTGATTGAAGCGATTTCCTTGGAAGAA GGTTTACAGCTCTTTGAAGGAGAGAGGGGATGCGTTGGTGAAAATCAA CAGTGTGCAGATTGGGCTGGTCCCCACTGCTGCTCAGGATATTATTGC ACTTGTCGATACTTTCCAAAATGCATATGTAGGAAAGACAGTGGAAAGT AATCTTCAATTTCATATTATTACCAACATTAAAAATGCAATATCAAACAG GAATGTGTATTAAAATTTTGTTTCATACCACACAGTTTTGTATTTCTGCT CAATTCAATAAAATATAATGAAATTTT	AY681336	mu-2Aaga_12
SEQ21	AACAAAATAACCATGAGAACTATCATTTCTCTGTTACTTCTTTCTGCTAT GGTCTTCGCCGTGATTGAAGCGATTTCCTTGGAAGAAGGTTTACAGCT CTTTGAAGGAGAGAGGGGATGCGTTGGTGAAAATCAACAGTGTGCAGA TTGGGCTGGTCCCCACTGCTGCTCAGGATATTATTGCACTTGTCGATAC TTTCCAAAATGCATATGTAGGAAAGACAGTGGAAAGTAATCTTCAATTTC ATATTATTACCAACATTAAAAATGCACTATCAAACAGGAA	AY681336	mu-2Aaga_12
I_36F#14	CAATGAAAAAATATCTGTATTCAGTCGAAACCAGCGTTTATCCATTGTTA TTTCTGCAGATACAATAAGGTGGTTGCCTGCAACTGCAGTAGAATCCAT CA	AY681337	mu-2Aaga_13
I_36F#17	TGATGGATTCTACTGCAGTTGCAGGCAACCACCTTATTGTATCTGCAGA AATAACAATGGATAAACGCTGGTTTCAACTGAATACAGATATTTTTTTCA TTGATCAGTTGTACTGTGTATTTCTTTCACGTGTAATGTATGAAATTTTG CATATTCCAACAAAATATATTGAAATTTG	AY681337	mu-2Aaga_13
II_36F#20	TGATGGATTCTACTGCAGTTGCAGGCAACCACCTTATTGTATCTGCAGA AATAACAATGGATAAACGCTGGTTTCAACTGAATACAGATATTTTTTTCA TTGATCAGTTGTACTGTGTATTTCTTTCACGTGTAATGTATGAAATTTTG CATATTCCAACAAAATATATTGAAATTTGAATATG	AY681337	mu-2Aaga_13
II-32F#5	CATATTCAAATTTCAATATATTTTGTTGGAATATGCAAAATTT	AY681337	mu-2Aaga_13
M08F#21	CAATGAAAAAATATCTGTATTCAGTTGAAACCAGCGTTTATCCATTGTTA TTTCTGCAGATACAATAAGGTGGTTGCCTGCAACTGCAGTAGAATCCAT CA	AY681337	mu-2Aaga_13
29F#12	CTGTGATGGATACTACTGCAGTTGCATGCAACCACCTAATTGTATCTGC AGAAATAACAATGGATAAACGCTAATTTCAACTGAATACAGATATTTTTC ATTGATTAGTTGTACTTTGTTTTCCTCTTATGTGTAATGTATGAAATTTTA CATATTCCAATAAAATATATTTAAATTTG	AY681338	mu-2Aaga_14
M10F#2	TACCCCTCCCGGTGGTTGCTTTAGCTCAAGCAAAAGGAGACACGGACG ATAAATTGAGACCTTGACAGTACAATAGCGTGCAAAAATAAAAATAAATT TTTAAATGTAATAAACATCAGCATGTATGAACTTCAATAAAAATTATTTTC AACATTGC	AY681342	AgorTX_B7a
29F#15	CTTAATTGTAAATTAATTTATTGTACAAAAGTTCTCCATCATCAGTAATTC TTTGCAATTATGCCATCTTTTTTTGCTAACCTAGTAATA		
29F#16	ACTGGGAAACAATATTTTTATTACCTAATTATAATAAATAAACTACAACAT ATTTACAGATATCAACAAATATATCTCAAAAGGTTGAGAAGTCAAATTAA GCAGCAGTGCATAATCGTTGCCTATATACAATTA		
I_19F#4	TTACCTTGCCCAACAACGAAAAGATTTTTTGGATCAGTCTCATCAGGTC TTTCATCGAAATAGGCCGGTGTTCACCGGCACCGCACTTCGGCGCCGG CACTAAGCTTTGTTGTCGGCGGCCAGCACGCACGAGTCTCGCCCAAAC CA		
II_19F#15	CATGTGCGTTTCACAAAACTTAACAAAAGGTGCAAAGTTTACAGTAAAA TATAACAGAATTGGTGCATTATAAAGGATTCCAAGAACAATATCTTGTAT AGAATATGAGAAAAGGTAAGTAAAATTTTATATCCTTTATGAACTTAATG AATTTTTTTTTCACACAAGCCAACATTAATATTT		

II_19F#2	CGTTAATTCATTCCTCATCGTGTTTAACTGAACGCTTTGTCATGATCAGA<br>TTTAAAATTGTCAAAAAACATTACTGCTGTAATTTGTTTACACTTTTTGCC<br>TTAAATAAAATATTAGGCATTC		
II_19F#5	AATCCTGGGTTGTTTTCAGTACTACTCATTCACAGAATTTTGTTAATTTG<br>CAGTATATTCAGTTTTCTTCAAAAAATTCAAAATTTCTGTGCTTGCAATAA<br>CCACATGTATCTAACCTGCCAGATAAATTTGATTTTGGCACTATTGCAAA<br>AAAGTAGTTCTGAAAGAGCTTCAAT		
M08F#14	TTAGTTTGTGTGTCAATATATTTTTGTTTTATGAAGAAGGCCTAGTTAAA<br>TGTGTAACTTTGGTAGTGGCAAAACCTCAATTTGTAATGTTAAATTGATA<br>AAAGTTTTAGTTTTTTCCACATCTATCATCGCTATTGTTCTGAATTGATCT<br>CGTTCTATAAAAGAACTTCTCTCCACTTTGTAACCAATCCCAGAAGTCTT<br>TCCACTGTTTGTAAAATAGAAAAAGGTATTTATGATAAATTATAGAAAAA<br>TTAATGTTGTTACACCTGTGAACAAGTGTATATAAGGCTATGTATCTTGA<br>AAACAAACCTGTTAATTAATAAAAAATGTTACC		
M08F#20	AAAAGATTAATTCTGGGATTTTATTACAAAAAATCAATAATTGATGATTTA<br>CATAAAATCTGCAAAATTGTTTATCTTATGAAATAAATACATGAATTTTAC<br>TGAAAACCACTAAAATGATCTGAATGAAACACTGCTTCTGAAAGCAGCA<br>AATTCCGTCAGTTTCGATGGATAACATAAATTTTTAGATATAACTTTTAG<br>AAAATTTTGTTAACATTAATCTTCTATCAGGTTGATGAAAATATGATGAAT<br>TATTTACAGGATGTACCATATGTTCACCTT		
M08F#5	ACAATATGCAATAGGCTTGAAATTTATAATTTTAGTCGATTACTTTAAAAA<br>GACCTTAGACCTACTTAATATTACAAATTTTGGATTTTTAGATTTTTCCCC<br>TTAATAGCGGCCCATAGGCTAAGCCCATACCAAATTTCAAGTCTGTAGG<br>CCCAGTAGATTGGGCTGGGAATTTCTATCACTCACT		
M10F#13	TAGGTGGTCTGTAAAAGGTTTCATAATTCTCTGCCATGAGTCAGTCTGA<br>AACTTTCAAGTCTTACCCTCGGTAAAGAATTAAAGCTAAATAAAATATGT<br>CTGGAGC		
M10F#19	GGGGTTTATAATTTTATTTGAAGGAAAATTACAAGAAAACATTTTTGAAT<br>CAAGTGAAACTACCTAGATTCTGATGAAACGAGAACCAGCTCATTCATA<br>CACCCGTCACA		
M10F#5	ATTTTATTTAGCTTTAATTCTTTACCGAGGGTAAGACTTGAAAGTTTCAG<br>ACTGACTCATGGCAGAGAATTATGAAACCTTTTACAGACCACCTA		
M11F#1	GAAGTGTTGAGAATGTTATTACTAATTTTTCCTACTCCTTACAGTCTCTG<br>ACAG		
M11F#12	GGCCCATTTTAGAGTATTGTATTTTTTAATATATATTATGTTTTGATTTTT<br>TTATGAAGTTCAAGATTTAGTAATGTTT		
M11F#16	ACAATCTTAATCACATCTTTATTATGGGCTCTGAACTTTTTTTTTTACATA<br>AATAAAATGTTTTCTTAATATATAGTTTTCATGTGGATTTGAATTCACAGA<br>ATAACATTCCAATTTAAACACTTCTTATGCAAATAAAGTTTCATTATACAC<br>AAAAATAGGAAATGTTTCAAATGGAAAGTGTTGCAGCAGCAGGAACAAT<br>ATATTGAAACATTTAATGTGCAGGGTTGAAGATGAGATTTGGCAGACTC<br>CAGATGCAACTTTTATGAACCCACAATAATATTGTTGATAGGAATATGAA<br>TAAGCTTGACAAAGAAACCAATAAAT		
M13F#1	CATTATTTAACATATAATTTATTTATTTATTACATTTTCTAGGATCCAATTA<br>TTTATGAATATAATTTAAAAACATGATCTCTTTTACATTCTCTTAAATGAA<br>GAATTGGATGTAGTAAAACAATCTATTCTTCGGAATATAACATGGTATTT<br>ATTAAGTTTTCATTGGCACGCCCGTTTATCCACGGGTCAACAGTTTGCC<br>CTTTTATCCACGGGTCCACAGCTCGCCCTTTAATCCACGGGTCCACGG<br>CTCGCACTAGAGGTTTGGGGAAATCGTTTCCTTCTATCTTATTTGGAAT<br>GGATCTTATATTTTCCACTGATTCCTCCCGCTTTTTATCGCCGCAGTCT<br>GGTCTAGGACATTGTCTTATATTTTCCACTGATTCCTCCCGTTTTTTA		

M13F#14	GGCATAATTTAACAAAAAATTTATTTATTTATAACATTTCCAAGGATCCAA<br>TTATTTTGGAAAATAATTTAAAAACAGGATCCCTTTTACATTCCCTTAAA<br>GGAAAAATTGGAGGTAGAAAAACAACCAATTCTTCGAAAAAAAACAGGG<br>AATTTATTAAGTTTTCATGGGCCCCCCCGTTTATCCCCGGGCCAACAGT<br>TGGCCCTTTTATCCCCGGGCCCCCAGCTCGCCCTTTAATCCACGGGCC<br>CCCGGTTCGAACAAAAGGTTGGGGAAAACCGTTCCCTTCAACCTAATT<br>GGGAAGGGATCTAAAATTTCCCCCGGATCCCCCCCGCTTTTTATCCCC<br>CCAGCCGGGCCAAGAAAATTGCCTAAAATTTCCCACGGATCCCCCCCG<br>TTTTTTA		
M13F#15	TAAATATGAGTGCAAAAATCAGAGATGCACAGCACGAAGGAACTGAGA<br>AAAATTCCTGATACGTATAACATGTAACGTGTACATCATAGTATTTGAAT<br>AAAATGTAATGTAATCTC		
M13F#16	GAGATTACATTACATTTTATTCAAATACTTAGATGTACACGTTACAAGTT<br>ATATGTATGAAATATTTCGCTCAATTCATCTATGCTGTGCATCTCTCATC<br>TATGCACTCATAATCA		
M13F#17	TTAAATAATGGAACAGTTGCTGTAGAGGAAGATATGTTGCTACATGTGA<br>TCCTCTTTTACTGTTGAACACTGTTCTAGAGCACACATCTATCCCCTCCC<br>TCCCCCCCCCCCTTTTATATTTTAAAATTATGTTTCTGTGCTTCAAATGC<br>AAAATTTAGGTTGTAAAAGCAAATTATTTTTAATAACTTGTGTACTGTGTA<br>GGTAGCTGGTAGAAATTTTTCATTGTGGAGAAGATTGTTTCAGATTACC<br>ACATGGAACCAACAATATTGTGGTAGTAAGGGGTTTATAAAATCAGG		
M13F#19	AAGGAACTATATTGACAGGATAATTTATTGTTAACAGCTTTACAGAATTT<br>AACATCCAGTGCTAAACATTTTAAACAGAAGTATTTTGATAACATTAGAT<br>GAAACAGAAATGTTTAATGCAGGTTTCAGCCAATTTTGAAGTCTATGAA<br>AATAAGAGGCCTTTAAATAGGCTAAACAGAAGGAACGTTTAACCATCCC<br>TGGTTTTCATGTACTTGATAAGATCAACTACTCTGCAACTGTATCCATAC<br>TCATTGTCATACCAGGAGACTAACTTCACAAAATTGTTGTTCAAGGAAAT<br>TCCAGCTTTGGCATCAAAAATACTGCTGTGAGAATCACCAATGAAGTCG<br>CTTGAAACAACCTCATCCTCTGTGTAGCCCAATATGCCTTTCCAGTTGT<br>CACTTTCGGAAGCAGCCTTAATAGCAGCTTTAATTTGATCATAGGTAGC<br>TTCTTTGGAAAGACGACAAGTCAAAT		
M13F#2	CATTGCAGTAATTCCTTTCAAGTTGCACTCGCAAAAGATGAACACACTT<br>CACTATTTATCGTGATATTAACGCGCACACTGTTCCACATTTGATGAAAT<br>TTCACAATTAAAATATTGATCACAAATCCACTTAACTTTTTCTTTGTAGAT<br>GCAGCAGGACGATCAGAAACAAGAATTATTTGTAGAAA		
M13F#21	TAAAAAACGGGAGGAATCAGTGGAAAATATAAGACAATGTCCTAGACCA<br>GACTGCGGCGATAAAAAGCGGGAGGAATCAGTGGAAAATATAAGATCC<br>ATTCCAAATAAGATAGAAGGAAACGATTTCCCCAAACCTCTAGTGCGAG<br>CCGTGGACCCGTGGATTAAAGGGCGAGCTGTGGACCCGTGGATAAAA<br>GGGCAAACTGTTGACCCGTGGATAAACGGGCGTGCCAATGAAAACTTA<br>ATAAATACCATGTTATATTCCGAAGAATAGATTGTTTTACTACATCCAAT<br>TCTTCATTTAAGAGAATGTAAAAGAGATCATGTTTTTAAATTATATTCATA<br>AATAATTGGATCCTAGAAAATGTAATAAATAAATAAATTATATGTT		
M13F#4	AACATATAATTTATTTATTTATTACATTTTCTAGGATCCAATTATTTATGAA<br>TATAATTTAAAAACATGATCTCTTTTACATTCTCTTAAATGAAGAATTGGA<br>TGTAGTAAAACAATCTATTCTTCGGAATATAACATGGTATTTATTAAGTTT<br>TCATTGGCACGCCCGTTTATCCACGGGTCAACAGTTTGCCCTTTTATCC<br>ACGGGTCCACAGCTCGCCCTTTAATCCACGGGTCCACGGCTCGCACTA<br>GAGGTTTGGGGAAATCGTTTCCTTCTATCTTATTTGGAATGGATCTTATA<br>TTTTCCACTGATTCCTCCCGCTTTTTATCGCCGCAGTCTGGTCTAGGAC<br>ATTGTCTTATATTTTCCACTGATTCCTCCCGTTTTTTA		
M13F#9	GTCTAAATGAATTAAAGACTGCCAAAGGAGAAGTAGTATCGAATTTATTT<br>TGAAATTTCGGAGGATTAAAATTATTCTGTTTATCTCATTATAGATATTTG<br>TAAACACGTTGGTTAATTTACCCAACGGAAATAATGCAAAATATTTATTA<br>CAAATAAATTTTTAATTAC		

This document was created with Win2PDF available at http://www.daneprairie.com.
The unregistered version of Win2PDF is for evaluation or non-commercial use only.
